# The Emerging Oncogenic Role of RARγ: From Stem Cell Regulation to a Potential Cancer Therapy

**DOI:** 10.3390/ijms26094357

**Published:** 2025-05-03

**Authors:** Geoffrey Brown

**Affiliations:** Department of Biomedical Sciences, School of Infection, Inflammation, and Immunology, College of Medicine and Health, University of Birmingham, Edgbaston, Birmingham B15 2TT, UK; g.brown@bham.ac.uk; Tel.: +44-(0)121-414-4082

**Keywords:** retinoic acid receptor γ, cell development, normal stem cells, cancer stem cells, carcinoma, therapeutics

## Abstract

Retinoic acid receptor (RAR) γ expression is restricted during adult haematopoiesis to haematopoietic stem cells and their immediate offspring and is required for their maintenance. From zebrafish studies, RARγ is selectively expressed by stem cells and agonism in the absence of exogenous all-*trans* retinoic acid blocked stem cell development. Recent findings for the expression of RARγ have revealed an oncogenic role in acute myeloid leukaemia and cholangiocarcinoma and colorectal, head and neck, hepatocellular, ovarian, pancreatic, prostate, and renal cancer. Overexpression and agonism of RARγ enhanced cell proliferation for head and neck, hepatocellular, and prostate cancer. RARγ antagonism, pan-RAR antagonism, and RARγ downregulation led to cell growth which was often followed by cell death for acute myeloid leukaemia, astrocytoma, and cholangiocarcinoma as well as hepatocellular, primitive, neuroectodermal ovarian, and prostate cancer. Histological studies have associated high level RARγ expression with high-grade disease, metastasis, and a poor prognosis for cholangiocarcinoma and ovarian, pancreatic, and prostate cancer. RARγ is expressed by cancer stem cells and is a targetable drive of cancer cell growth and survival.

## 1. Introduction

The transformation of a normal cell into a malignant cancer cell involves at least two discrete steps, with the first giving rise to a pre-malignant clone and the second conversion to overt malignancy [1]. Changes to the genome via oncogenes lead to a dominant gain of function, and disruption to tumour suppressor genes leads to a recessive loss of function. The molecular machinery that regulates cell proliferation, differentiation, and survival versus death is similar in all mammalian cells. Therefore, changes to the genetic programme of cancer cells have highjacked the normal control circuitry, and they often endow cancer cells with a selective growth advantage, or a lower probability of cell death compared to normal cells [2,3]. Cancer cells may also be described as ‘anarchistic’ regarding their lack of compliance with normal behaviour controls, and by contrast, normal cells behave in a ‘social’ manner that benefits an organism. In 2000, Hanahan and Weinberg listed the characteristics that are shared by most cancers as an ability to sustain proliferative signalling, evade growth suppression, resist cell death, enable replicative immortality, induce angiogenesis, and activate invasion and metastasis [4]. The abilities to reprogram energy metabolism and evade immune destruction and attention to the importance of the tumour environment were added in 2011 [5]. Efforts to develop effective cancer treatments are driven by looking to kill cancer cells and spare normal cells as much as is possible.

To return to normal tissue cells, they are organised as a hierarchy and, as such, are a heterogenous population that includes self-maintaining stem cells, cells that amplify the production of mature cells from stem cells, and functional end cells that are dying. The cancer stem cell theory states that cancers also display the above feature of normal tissue organisation whereby a small subset of cancer stem cells (CSCs) generates a hierarchy of cells that are the bulk of the cancer cells [6]. It is also important to bear in mind that the generation of CSCs may often involve the transformation of a tissue stem cell [7], and CSCs then inherit features of a normal stem cell, particularly an unlimited growth potential. Whereupon a tissue stem cell is transformed, the molecular controls of interest regarding the abnormal behaviour of cancer cells may lie in part within a change(s) to events that particularly control normal stem cell behaviour. In other words, the maintenance of the CSC compartment and/or their nature as compared to normal stem cells may be deregulated.

The retinoic acid receptor (RAR) γ is selectively expressed by normal stem cells, and expression is needed for their maintenance. RARγ is an oncogene for many different cancers, including acute myeloid leukaemia and cholangiocarcinoma, and colorectal, head and neck, hepatocellular, ovarian, pancreatic, prostate, and renal cancers. A transformation-provoked change to the normal control circuitry is often due to the overexpression of an oncogene. RARγ is overexpressed in the above cancers other than in acute myeloid leukaemia, which harbours a RARγ fusion protein. Ectopic overexpression of RARγ in cancer cells enhances cell proliferation, and downregulation reduces cell proliferation. This review examines the influence of RARγ on the behaviour of normal and cancer cells and the proposed oncogenic roles of RARγ.

## 2. RARγ Is Expressed by Normal Stem Cells

The restricted spatial and temporal patterns of expression of RARγ transcripts seen from mouse embryogenesis studies suggested a role for RARγ during early morphogenesis, chondrogenesis, and squamous epithelial differentiation. Support to a role within stem/progenitor cells and an involvement in tissue development was as follows. RARγ transcripts were detected at day 6.5 post conception (p.c.), when mesoderm developed from the primitive streak, and at day 8 p.c. in all germ cell layers in the posterior embryo region. Regarding chondrogenesis, high levels of transcripts were still found in frontonasal and pharyngeal mesenchymal derivatives at day 12.5 p.c. From day 13.5 p.c., RARγ transcripts had accumulated in neural ectoderm and endoderm derivatives and when epithelia had started to differentiate. RARγ transcripts were found in the roots of the developing whisker follicles and the regions where the teeth develop [8].

Studies of zebrafish embryos support the view that RARγ is expressed by the stem/progenitor cells to tissue development. Zebrafish embryos express two major RAR paralogs, namely RARα and RARγ. There are two paralogs of RARγ, namely RARγa and RARγb. They are paralogs rather than splice isoforms because the *RARγa* gene is located on chromosome 23 and the *RARγb* gene on chromosome 6. *RARγb* gene expression was seen to be expressed by the three streams of migrating cranial neural crest cells and at a high level in the anterior and tailbud regions [9]. Other work reported strong expression of the *RARγ* gene in the posterior hindbrain at late somitogenesis, in all three neural crest streams and the neural crest-derived mesenchymal cells that will occupy the anterior pharyngeal arches, and in the tail [10]. Expression of the *RARγ* gene is, therefore, restricted to mesodermal and neural crest stem/progenitor cells in the head area, the lateral plate mesoderm, and the presomitic mesoderm of the tail bud. Earlier studies had shown that all-*trans* retinoic acid (ATRA) signalling was essential for patterning the central nervous system anterior–posterior axis and inducing a pectoral fin bud [11]. For Xenopus laevis embryos, RARγ2 was observed at the gastrula and neurula stages, in the head endomesoderm, the neuroectoderm of the hindbrain region, and the entire posterior region. From these findings, the investigators concluded that ATRA has a direct action via RARγ2 on the developing head mesoderm and posterior neuroectoderm [12].

Expression of the major isoforms of RARα, β, and γ has been mapped out for developing haematopoietic stem cells (HSCs). This was undertaken for a population of mouse cells that contained HSCs (lineage^−^, c-kit^+^, and Sca-1^+^ (LKS^+^) cells) and a cell population that did not contain HSCs (lineage^−^, c-kit^−^, Sca-1^−^ (LKS^−^) cells). Both LKS^+^ and LKS^−^ cells expressed RARα, and LSK^+^ cells preferentially expressed RARβ2 and RARγ1 [13]. Hence, expression of RARγ and RARβ2 was restricted to HSCs and their immediate offspring. Expression of RARα, particularly of the RARα2 isoform, increased dramatically when the factor-dependent cell-Paterson (FDCP)-mixA4 murine progenitor cells were induced to undergo myelomonocytic differentiation [14], and it is well known that RARα plays a role during neutrophil differentiation [15].

## 3. A Role for RARγ During Embryogenesis and Adult Haematopoiesis

Mouse embryonic stem cells express RARα and RARγ proteins, and ATRA-regulated expression of the clustered *Hox* genes is required to establish the anterior–posterior body plan during embryogenesis [16]. Studies that made use of mutant embryonic cell lines that were deficient in RARγ showed that RARγ plays a critical role in the epigenomic reorganisation of the *Hox* gene clusters via interactions with distinct enhancer elements. RARγ was essential for ATRA-induced *Hoxa1* transcription activation because deletion of the RARγ binding site in the *Hoxa*1 gene enhancer attenuated the epigenomic and transcriptional activation of the *Hoxa* and *Hoxb* gene clusters. Complete erasure of the polycomb repressive mark H3K26me3 was not required for *Hox* gene activation. However, all-trans retinoic acid (ATRA) activation of RARγ was required for the erasure of H3K26me3 from activated *Hox* genes during embryonic stem cell differentiation. The investigators proposed that an RARγ-dependent activity permits the initiation of transcription by unharnessing preassembled complexes [16].

From studies of ATRA signalling in the marine annelid Platynereis dumerilii, the investigators proposed that ancestral RAR is a low-affinity sensor that triggered neuronal differentiation [17] and ATRA enhances the differentiation of embryonic stem cells into neuronal precursors [18]. *Hoxa1* gene expression was required for embryonic stem cells to adopt a neuronal fate because Hoxa1^−/−^ embryonic stem cells failed to differentiate along a neuronal lineage when treated with ATRA [19]. As mentioned above, RARγ is needed for *Hoxa1* gene transcription activation, and RARγ has been proposed to play a role in embryonic stem cells adopting a neuronal lineage whereby ATRA activation and receptor phosphorylation are both essential [20]. From studies of undifferentiated F9 embryonal stem cells, RAR/RXR dimers bound to genomic sites that are important to stem cell stemness because the sites were also targeted by transcription factors that maintain pluripotency, including SOX2, NANOG, and POU5F1. Dimers with retinoid X receptors can also distinguish between pluripotency- and differentiation-associated cis-regulatory elements [21].

The ligation status of RARγ is important to stem/progenitor cell development as follows. For zebrafish embryos, the development and growth of tissues derived from cranial neural crest and primitive mesoderm were blocked by agonism of RARγ. Zebrafish embryos were treated at 4 h post fertilisation with 80 nM of the selective RARγ agonist AGN205327 in the absence of exogenous ATRA. This led to severe truncation, and RARγ agonism prevented the formation of the most posterior somites. The formation of tissues arising from cranial neural crest, including cranial bones and anterior neural ganglia, was disrupted. Pectoral fine growth was blocked by the agonist. The Tbx-5-immunopositive lateral plate mesodermal stem/progenitor cells were intact because the RARγ agonist block was reversed by replacing the medium with a control medium (washout) or medium that contained 10 nM of the RARγ antagonist AGN205728 at 27 h post fertilisation. The addition of the RARγ agonist also blocked caudal fin regeneration when the fin was transected at 2 days post fertilisation. Again, the block was reversed by washout at 24 h post-transection and by the subsequent addition of the antagonist. Treatment of *hoxb13a* reporter zebrafish embryos with the RARγ agonist revealed an association between a complete loss of *hoxb13a* gene expression and a lack of caudal fin formation. Hoxb13a expression was rescued by washout of the RARγ agonist, and subsequent treatment with the RARγ antagonist and caudal fin length increased [22].

Interference with stem/progenitor cell development was also seen when mouse mesenchymal stem cells were treated with RARγ agonist NRX204647. Treatment of micro-mass cultures of mesenchymal embryo limb cells, isolated on embryonic day 11.5, with the RARγ agonist, blocked the formation of cartilaginous nodules. When mesenchymal stem cells were treated with the RARγ agonist and transplanted into nude mice, they did not give rise to ectopic bone masses as seen for control cells. The treated cells had become unresponsive in vitro to recombinant bone morphogenic protein-2 and had either lost skeletogenic potential or this potential had been blocked. The RARγ agonist also blocked heterotopic ossification in a transgenic mouse model that expressed a constitutively active kinase that was like the activin receptor-like kinase-2 that is seen in fibrodysplasia ossificans progressiva [23].

Findings from studies of the role of RARγ during Xenopus embryo development also emphasise the importance of the ligation status of RARγ. These studies made use of the RARγ-selective agonist NRX204647, a constitutively active RAR (VP16-RARγ2), and overexpression of a dominant-negative co-repressor (c-SMART). They showed that active repression by RARγ signalling was necessary for vertebrate axial elongation. Moreover, the investigators argued that RARγ played roles at all stages of axial elongation, and the different roles were related to the ligation status of RARγ. They proposed that RARγ2 functions as an activator near the determination wavefront and subsequently as a repressor to maintain progenitor pools in the presomatic mesoderm and chordoneural hinge. In modelling the role of RARγ, they also argued that when the progenitor pool becomes exhausted as axial elongation nears completion of the RARγ2 function as an activator to terminate elongation. In this case, RARγ2 promotes apoptosis to terminate elongation by virtue of ATRA coming into proximity to the caudal domain RARγ2. The model emphasises that the proximity of ATRA to RARγ2 is important to whether RARγ2 is active or inactive, and, in turn, this is important to whether the required context to the role of RARγ2 is to control either gene expression or repression. These findings bring to attention that cell context and the ligation status of RARγ are both important to the nature of the action of RARγ during development [24].

From studies of the RARγ knockout mouse, RARγ expression was needed for the maintenance of HSCs. The number of HSCs was markedly reduced in the bone marrow of RARγ knockout mice. Compared with wild-type mice, increased numbers of mature progenitor cells were seen, which may have been related to the loss of RARγ regarding the ability of agonised RARγ to inhibit stem cell development (see above). ATRA promoted the maintenance of HSCs in culture, as seen from transplantation studies, and loss of RARγ abrogated this potentiating effect. HSC abnormalities were not seen for RARα knockout mice, and the bone marrow cells from these mice were able to self-renew in culture in response to ATRA treatment. Additionally, primitive hematopoietic precursors that overexpressed RARγ exhibited a substantially more undifferentiated phenotype. Therefore, RARγ has a distinct role in hematopoiesis which is to regulate HSC self-renewal/stemness by presumably influencing the balance of decision-making by HSCs in favour of self-renewal as opposed to differentiation [13]. During hematopoiesis, RARγ and RARα play reciprocal roles in promoting cell self-renewal and myeloid differentiation (see above), respectively.

## 4. RARγ Plays a Role in Cancer

RARγ is an oncogene for many cancers, including AML, cholangiocarcinoma, colorectal, head and neck, hepatocellular, ovarian, pancreatic, prostate, and renal. Table 1 provides an overview of the findings that support this view. RARγ expression is downregulated by the microRNA 30a-5p [25], and this microRNA is a tumour suppressor that is expressed at a low level in many cancers [26]. As mentioned above, RARγ is required for epigenomic marks, and there is support for the view that RARγ plays an oncogenic role in this manner. There is also support for RARγ activation of Akt/NF-kB and Wnt/β-catenin signalling pathways. Accordingly, RARγ has been reported to be seen predominantly in the nucleus, for example, in prostate cancer, or in the cytoplasm, for example, in hepatocellular cancer. It seems that RARγ can move from the cytoplasm to the nucleus and vice versa.

### 4.1. AML

In some acute promyelocytic leukaemia patients, there are rearrangements of the *RARG* gene rather than of the *RARA* gene. The patients with *RARG* gene rearrangements did not respond to ATRA-based therapy other than a patient with a *PML-RARG* translocation [27]. A later study by other workers examined the most prevalent *RARG* gene fusion and showed that CPSF6-RARγ interacts with histone deacetylase 3 to promote myeloid transformation. The fusion enhanced the expansion of myeloid progenitors, their differentiation was impaired, and interaction of the fusion with histone deacetylase 3 suppressed gene expression that was associated with myeloid differentiation, including expression of the transcription factor PU.1. More aggressive myeloid malignancies were driven by a synergy between the RARγ fusion and RAS mutations [28].

### 4.2. Cholangiocarcinoma

RARγ was seen to be frequently overexpressed in patients’ cholangiocarcinoma specimens, and high expression was associated with lymph node metastasis. The proliferation, migration, invasion, and colony formation ability of cholangiocarcinoma cells in vitro and the tumorigenic potential of cells in nude mice were reduced by the downregulation of RARγ. In response to knockdown, expression of the cell cycle inhibitor P21 was upregulated, and the levels of cyclin D1, proliferating cell nuclear antigen, and matrix metallopeptidase 9 were downregulated. Suppression of the Akt/NF-κB pathway was observed in parallel. RARγ overexpression contributed to the multidrug chemoresistance of cholangiocarcinoma cells, which, in part, was related to P glycoprotein upregulation via activation of the Wnt/β-catenin pathway. From these findings, the investigators concluded that RARγ plays an oncogenic role in cholangiocarcinoma cells via activation of the Akt/NF-κB and Wnt/β-catenin pathways [29].

### 4.3. Colorectal Cancer

For human colorectal cancer specimens, RARγ was frequently overexpressed and mainly resided in the cytoplasm. Knockdown did not affect colorectal cancer cell proliferation nor block cell cycle progression. It increased the sensitivity of cells to chemotherapeutics, and this was attributed to the downregulation of multidrug resistance (MDR1) and suppression of the Wnt/β-catenin pathway. For tissue specimens, a positive association was observed between RARγ and MDR1 [30]. Other workers have described a signature for colorectal carcinoma brain metastases that is associated with poor patient survival and brought to attention that vitamin A metabolism might be a regulator for metastatic brain tropism. For central nervous system-resident metastases, they observed upregulation of the expression of DHRS9 and CYP26B1. DHRS9 catalyses the first step in ATRA synthesis, and CYP26B1 catabolises ATRA [31].

### 4.4. Head and Neck Cancer

The predominant RARγ 1, 2, and 3 isoforms are expressed by head and neck cancer cells. Ligand activated RARγ accelerated the cell cycle progression of head and neck cancer cell line cells, and this required CDK7-dependent phosphorylation of RARγ. shRNA knockdown abolished the proliferation of head and neck, oesophageal, breast, and colon cancer cell line cells and prevented the tumour formation in nude mice by xenografted head and neck, lung, and colon cancer cell line cells. RARγ interacted with vinexin-β, and ligand activation of RARγ resulted in activation of the epidermal growth factor receptor and downstream Akt, Erk, Src, and YAP signalling [32].

### 4.5. Hepatocellular Cancer

Most human hepatocellular carcinoma tissue samples and hepatocellular carcinoma cell lines had significantly elevated levels of RARγ, and RARγ was seen to be mainly in the cytoplasm. Colony formation by the cell lines in vitro and the growth of xenografts of the cell line HepG2 in nude mice was promoted by overexpression of RARγ. RARγ was observed to interact with the p85α regulatory subunit of phosphatidylinositol 3-kinase (PI3K), which led to the activation of Akt and NF-κB signalling pathways. From these findings, the investigators concluded that oncogenic RARγ plays a nongenomic role in promoting cell survival and growth [33]. Other workers have reported that upregulated expression of RARγ in human tissues correlates with tumour size and distant metastases and negatively with patient survival. They reported a negative correlation between RARγ expression and expression of E-cadherin and that RARγ downregulation of E-cadherin is mediated by NF-κB [34].

### 4.6. Ovarian Cancer

A high level of expression of RARγ mRNA was associated with FIGO stage III/IV ovarian cancer and proposed as an independent predictor of poor overall survival. The level of RARγ protein was also higher in ovarian cancer tissue than in adjacent normal tissue. For ovarian cancer cells, high expression of RARγ accelerated cancer progression by promoting cell proliferation. From in vitro studies, the proliferation and colony formation capacity of ovarian cancer cell line cells were suppressed by downregulation of RARγ, and tumour growth by cell line cells in nude mice was significantly reduced by knockdown of RARγ. Decreased levels of expression of Ki-67 and proliferation cell nuclear antigen were observed [35].

### 4.7. Pancreatic Cancer

Overexpression of RARγ is common in human pancreatic cancer and predictive of a poor patient prognosis. RARγ was required for the proliferation of pancreatic cancer cells because knockout of *RARG* expression reduced the proliferation of the PANC1, BXPC3, and Sw1990 cell line cells in vitro, with the cells arresting in the S phase of the cell cycle. Knockout also reduced tumour growth by PANC1 cells in NOD-SCID mice. RARγ was shown to bind directly to the promotors of MYC, STAT3, and SLC2A1, and this had most likely led to chromatin epigenetic activation from the use of ChIP-qPCR and measurement of the deposition of the gene activation marker histone H3 K27 acetylation (H3K27ac). The deposition of H3K27ac was reduced in *RARG* knockout cells [36]. Other workers have reported that overexpression of RARγ is associated with a poor patient prognosis and that siRNA knockdown induced the arrest of pancreatic cell lines in the G1 phase of the cell cycle but that this did not lead to apoptosis [37].

### 4.8. Prostate Cancer

RARγ and RARα were increased significantly in high-grade prostate cancer, and a moderate to strong intensity of RARγ was seen in the nuclei of high-grade cells [38]. Treatment of human prostate cancer cell lines with the RARγ agonist AGN205327 stimulated cell proliferation [39]. RARγ plays a role in the cytoarchitecture of the prostate because RARγ-Foxa1 has been shown to promote luminal identity in adult mouse prostate progenitors. From studies of prostate organoids that were established from normal mice, ATRA activation of RARγ promoted expression of Foxa1, which synergised with the androgen pathway, via reshaping of the binding of the androgen receptor genome-wide to ensure proper luminal expansion and cytoarchitecture. Treatment of the organoids with the RARγ antagonist LY2955303 reduced lumen formation. *FOXA1* mutations are common in prostate cancer, and the investigators showed that cancer-associated and loss-of-function mutations affecting residue F254 promoted the dedifferentiation of adult prostate progenitors. From these findings, the roles of RARγ and FOXA1 in prostate cancer deserve close attention by means of orthotopic transplantation of normal and tumour organoids [40].

### 4.9. Renal Cancer

The levels of mRNA for RARγ and RARβ were upregulated in clear renal cell cancer from bioinformatics analysis and the use of quantitative PCR, and the investigators proposed that these changes may contribute to disease progression [41].

## 5. RARγ Ensures the Maintenance of Normal and Cancer Stem Cells

**Figure 1:**
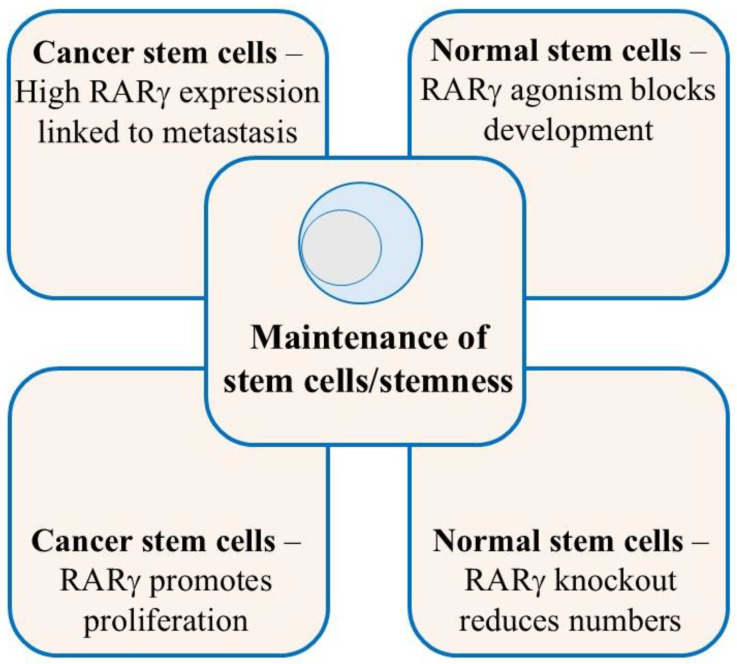
Key features to the actions of RARγ.

As seen for haematopoiesis, the presence of RARγ is important to the maintenance of HSCs and/or their stemness (Figure 1). The zebrafish embryo studies were undertaken in an E3 fish medium that lacked ATRA; all-*trans* retinol and RARγ agonism were sufficient to ensure stem cell maintenance, and their differentiation was blocked. It has been reported that embryonic stem cells do not have the capacity to metabolise all-*trans* retinol into ATRA. The cells examined could not transport all-*trans* retinol into the cytoplasm because of an absence of the specific cell surface receptor stimulated by retinoic acid 6 (STRA6). The all-*trans* retinol metabolising enzymes Adh4, Adh1, and RALDH2 were also absent [42,43]. Even so, we do not know for certain the extent to which normal stem cells can make ATRA because such has not been measured directly as this is difficult. If normal stem cells cannot make ATRA, the levels of ATRA in embryonic tissues are tightly controlled, and they may just see ATRA at a low level, as provided by neighbouring cells. If normal stem cells can make ATRA, they may just do so at a low level. In both scenarios, normal stem cells may reside in a low ATRA environment in vivo. In this regard, RARγ is transactivated by ATRA concentrations below 10^−9^ M (EC_50_ = 0.39 × 10^−9^). Thirty-three-fold more ATRA was needed to transactivate RARα (EC_50_ = 12.9 × 10^−9^ M), and a maximal effect was seen as the concentration approached 10^−7^ M [44,45]. Again, cell context is important to the action of RARγ regarding whether the level of ATRA is sufficient to activate RARγ and not RARα. The purpose of RARγ may be to ensure the maintenance of stem cells when they are in a low ATRA environment, which would also protect stem cells from RARα-driven differentiation. Additionally, RARγ is a weak transcription activator as compared to RARα. Transactivation by RARα was 10-fold higher than that seen for RARγ, as revealed by the fold-induction of luciferase activity from a RARE-tk- Luc reporter plasmid in the presence of each RAR. This finding suggests a maintenance or gene priming role for RARγ [45].

The role of RARγ in maintaining normal stem cells extends to CSCs. This is highly likely to be the case if the CSC arose from the transformation of a normal stem cell. Patients’ cancer cells often reside in a low ATRA environment. The level of ATRA for prostate cancer tumours was measured at ~1 ng/g tissue which is near the limit of detection. Eight times more ATRA was observed for adjacent normal prostate epithelium cells and benign hyperplasia cells [46]. Increased cellular retinoic acid-binding protein-1 may have accelerated ATRA degradation because prostate cancer tumours have an increased level [47,48]. The concentrations of ATRA and all-*trans* retinol have been reported to be low in pancreatic ductal adenocarcinoma patients’ cells, and this was associated with a worse patient survival outcome [49]. Reduced levels of all-*trans* retinol and retinyl esters have been reported for patients’ renal cancer cells [50]. ATRA biosynthesis is deregulated in many cancers as reviewed in [26]. An absence of all-*trans*-retinol-metabolising enzymes has been reported for CSCs for two clones (KC1 and KC4) that were derived from mouse mammary tumours, and that gave rise to invasive and metastatic tumours. KC1 cells lacked Adh1, Adh4, and RALDH2 and KC4 cells lacked just Adh1 and Adh4 [51]. Low or no ATRA synthesis has been reported for breast cancer cell lines [52,53], and human ovarian cancer cell lines lack ATRA synthesis [54]. Expression of the enzymes required for ATRA biosynthesis has been reported to be low in human colorectal [55,56] and ovarian [57] cancer cell lines and patients’ gastric [58], lung [59], and head and neck [60] cancer cells. Cancer cells have either adapted to survive and grow in a low ATRA environment or may be just like their normal stem cell counterpart. In either case, they are dependent on RARγ signalling whereby targeting RARγ provides a means to growth arrest and kill cancer cells as follows.

## 6. Targeting RARγ to Kill Cancer Stem Cells

Various approaches have been used to interfere with the activities of RARs, including pan-RAR and selective RARγ antagonists and inhibition of the enzymes that are needed for ATRA biosynthesis. The use of enzyme inhibitors has been reviewed elsewhere [26]. Table 2 shows the findings from the use of pan-RAR and selective RARγ antagonists, whereby treatment of cancer cells leads to growth arrest and cell death. Some studies have antagonised all RARs, and therefore, it cannot be presumed that the effector target is RARγ. Even so, there is support for the view that antagonism of RARγ is sufficient to kill CSCs (see table below for prostate cancer).

### 6.1. AML

The pan-RAR antagonist AGN193109 inhibited AML cell stemness and leukemogenesis in vivo. These studies made use of Evi1^high^ and MA-9-driven mouse models of AML, and mice were transplanted with leukemic stem cells from terminally ill mice and then treated with AGN193109 for two weeks. Treatment improved overall survival as compared to vehicle control and decreased the percentage of leukemic stem cells in the spleen. From re-transplantation experiments, AGN193109 treatment of primary recipients led to a delay in leukemogenesis and a decreased spleen weight in secondary recipients. AGN193109 also decreased stemness in primary human AML samples. Samples from four EVI1^high^ and two EVI1^low^ AML patients were treated for three days with AGN193109 or ATRA. The level of CD11b expression by these primary cells was small. AGN193109 decreased the clonogenic capacity in 3/4 of the EVI1^high^ samples and had a minimal effect on the EVI1^low^ samples. ATRA increased the clonogenic capacity in 3/4 of the EVI1^high^ samples and slightly decreased the colony formation by the EVI1^low^ samples. Hence, AGN193109 had counteracted the stem/progenitor properties of patients’ AML cells, whereas ATRA had promoted [61].

### 6.2. Hepatocellular Cancer

The natural flavonoid acacetin was used to modulate the influence of RARγ on the AKT-p53 network. Acacetin specifically binds to RARγ to inhibit RARγ transactivation that is stimulated by ATRA, as was seen from reporter assays. The growth of the hepatocellular cancer cell lines HepG2, QGY-7703, and SMMC7721 was strongly inhibited by acacetin, and the Bel-7402 hepatocellular cancer cells and normal LO2 liver cells were seen to be resistant. The SW480 and SW620 colon cancer cells were unresponsive to acacetin. As mentioned above, RARγ was present in the cytoplasm of hepatocellular cancer cells, and acacetin induction of apoptosis was reported to be due to antagonism of a non-genomic action of RARγ whereby cells were switched from pro-survival to pro-apoptosis. Acacetin inactivated AKT by means of RARγ, and inactivation of AKT led to activation of Bax and, in turn, apoptosis [62].

### 6.3. Pancreatic Cancer

The RARγ antagonists LY2955303 and MM11225 suppressed the proliferation of pancreatic cancer cell lines by inducing G1 arrest, which was not followed by apoptosis. Treatment of the cell line cells led to upregulation of p21 and p27 and downregulation of cell cycle genes, including the cyclin-dependent kinases 2, 4, and 6. The proliferation of five established patient-derived pancreatic cancer organoids was reduced by the two RARγ antagonists [37].

### 6.4. Paediatric Brain

Primary cell cultures that were obtained from two paediatric patients with primitive neuroectodermal tumours and a patient with an astrocytoma were treated with the pan-RAR antagonist AGN194310. The CSCs that give rise to the immature and differentiating cells within neurospheres and the progeny of CSCs were ablated by treatment with the pan-RAR antagonist, and neurosphere formation was prevented. The pan-RAR antagonists were substantially more effective than ATRA and the RARα-selective agonist AGN195183. In these studies, the pan-RAR antagonist did not affect human fibroblasts and blood mononuclear cells [63].

### 6.5. Prostate Cancer

The pan-RAR antagonist AGN194310 and the RARγ antagonist AGN205728 were highly effective in driving growth arrest in the G1 phase of the cell cycle, followed by necroptosis of the LNCaP, DU145, and PC3 cell line cells and patients’ primary cells. Cell death was via necroptosis because, whilst mitochondrial-dependent, the process was caspase-independent and blocked by inhibition of the poly(ADP-ribose) polymerase PARP-1 [39,64,65]. Both compounds ablated the formation of colonies by the serum free grown cell line cells whereby the pan-RAR antagonist was effective at concentrations between 18 and 34 nM (IC_50_) and the RARγ antagonist AGN205728 at between 3 and 6 nM (IC_50_). Therefore, CSC-like cells were killed by the antagonists in addition to the non-CSC-like cells. For comparison, the IC_50_ values obtained for the prevention of colony formation by ATRA were between 344 and 402 nM. The RARα selective antagonist AGN196996 was less effective than ATRA in preventing colony formation by the three prostate cancer cell lines. These data support the view that antagonism of RARγ alone is sufficient to prevent colony formation by the prostate cancer cell lines. For the cell line cells, synergy was observed when they were treated with the RARγ antagonist AGN205728 in combination with docetal. RARγ is predominantly expressed by around 20% of basal and 30% of luminal proximal progenitors in the human prostate. As mentioned above, antagonism of RARγ reduced lumen formation by normal mouse prostate organoids [40]. This may relate to the exhaustion of the progenitor pool.

As RARγ controls the behaviour of both normal and cancer stem cells, it is important to consider the extent to which normal cells might be affected when targeting RARγ to kill cancer cells. Cancer cells appear to be more sensitive to the targeting of RARγ than normal cells. The pan-RAR antagonist AGN194310 was more effective against patients’ prostate cancer cells and cell line cells than normal prostate fibroblasts and epithelial cells [65], and the RARγ antagonist AGN205728 was more effective against prostate cancer cell line cells than the normal prostate RWPE-1 cells [39].

Are RAR antagonists safe to use in animals? A substantial dose of the pan-RAR antagonist BMS-189453 was given to mice and rats and inhibited spermatogenesis, and this was reversible upon withdrawal of the antagonist. In the mouse experiments, BMS-18953 was given at 2.5 mg/kg for 4 weeks. There were no changes in body weight, no gross or histological abnormalities in any tissues/organs from gross necropsy, and no changes in haematology and serum chemistry analyses [66]. In the rat studies, testicular degeneration/atrophy occurred at doses of 2 mg/kg for 7 days and 10 mg/kg for 3 days, and that produced no other toxicities [67]. Antagonising all RARs seemed to have little effect on the condition of the animals nor gave rise to abnormalities other than blocking spermatogenesis. These findings indicate that other adult stem/progenitor cell pools were not noticeably compromised and perhaps neither critically nor entirely dependent on ATRA availability. Even so, there is the need to treat mice and rats with antagonists for months to ensure that there is no substantial detriment to the stem cells that are replacing worn-out cells in tissues.

As considered above, ATRA activation of RARγ is needed for neuronal differentiation of embryonic stem cells. Studies that made use of selective RARα, β, and γ, revealed that RARγ was the functionally dominant RAR that mediates the development of stiatopallal-like neurons from embryonal carcinoma cells. Activation of RARα was less efficient in driving the development of these neurons [68]. Regarding the antagonism of RARγ, RARγ may be less functionally dominant to the maintenance of adult stem cells, whereby other isotypes can adequately mediate ATRA signalling. Treatment of purified human HSCs (Lineage^−^, CD133^+^, CD34^+^) with 100 nM of the RARγ antagonist AGN205728 did not affect cell proliferation in vitro, and treatment with the RARα antagonist AGN196996 enhanced proliferation [44]. CSCs are sensitive to RARγ antagonism, and it is interesting to speculate whether they share or have hijacked embryonic RAR-related molecular mechanisms and signalling pathways. Cancer cells can undergo deprogramming to the extent of re-expressing embryonic genes [69], and other workers have proposed that the reactivation of embryonic programmes plays a role in the initiation of cancer [70]. RARγ can play a role in programming the identity of cells. The four transcription factors Oct4, Sox2, Kif4, and C-Myc were used to reprogram somatic cells to induced pluripotent stem cells. The addition of RARγ and liver homologue 1 to the four factors led to rapid and efficient reprogramming of human neonatal and adult fibroblast cells to induced pluripotent stem cells that resembled mouse embryonic stem cells and that were factor dependent. The investigators proposed that RARγ and liver homologue 1 had directed reprogramming towards ground-state pluripotency [71]. In this case, RARγ appears to play a role in the establishment and/or maintenance of pluripotency. There is a need for a better understanding of the functional dominance of RARs in the context of stem cells and to compare the sensitivity of adult stem cells, embryonic stem cells, and CSCs to pan-RAR and RARγ antagonism.

## 7. Concluding Remarks

There is a need to find new treatments for cancer, particularly for disease relapse and aggressive and metastatic cancer. A focus of attention for some years has been to look for the means to eradicate CSCs for various reasons. In 1999, Holyoake and colleagues provided definitive evidence for the presence of a quiescent subpopulation of leukaemia cells in chronic myeloid leukaemia that had stem cell properties from in vitro and in vivo studies [72]. Subsequent studies showed that some of these quiescent cells were insensitive in vitro to STI571, which killed all dividing cells by means of its action against the p210^BCR-ABL^ tyrosine kinase [73]. There is growing evidence to support the view that the CSCs that are insensitive to the conventional chemotherapeutics that kill dividing cells are responsible for disease relapse, metastasis, and a poor prognosis [74].

From all the findings for RARγ, inhibiting its activity provides a promising approach to eradicating CSCs to address the problems of metastasis and disease recurrence. For example, some AML patients may benefit from antagonism of all RARs because leukemogenesis was delayed and stemness was reduced in an Evi1^high^ and MA-9 driven mouse model of AML by the pan-RAR antagonist AGN193109 [61]. Head and neck cancer patients may benefit because active RARγ isoforms promote head and neck cancer proliferation [32]. Findings from immunohistology studies have led to a link between high RARγ expression and aggressive and metastatic disease. Even so and yet unresolved is whether the oncogenic action of RARγ has led to a change in the behaviour of CSCs as compared to that of a normal stem cell or has changed a normal stem cell to give rise to CSCs. Also, in either case, what is the main importance to cancer?

There is much that needs to be unravelled about the actions of RARs in regulating gene expression that is germane to using RAR antagonists to manipulate the activities of RARs to control the behaviour of and/or kill CSCs. Whereby cells express RARα, β, and γ and RXRα, β, and γ, there are many possible dimer combinations, and they can interchange at gene loci. RARα and RARγ, and presumable RARβ, play different roles, and they also crosstalk with other nuclear receptors [75]. As mentioned above, RARγ modulates cellular signalling events and is subject to modulation, for example, phosphorylation by signalling events. Finally, it is important to bear in mind that the exposure of cancer cells to immune cells may be influenced by antagonising RARs because they, including RARγ, are pleiotropic modulators of the immune system as reviewed in [76]. Encouragingly, the pan-RAR antagonist has been shown to be effective against leukemogenesis and appears to be safe to use in animals. It seems that there is just the need to antagonise RARγ.

## Figures and Tables

**Table 1:** RARγ is an oncogene.

Malignancy	Evidence That RARγ Is an Oncogene
AML	*RARG* gene rearrangements [27]; CPSF6-RARγ interacts with histone deacetylase 3 to promote myeloid transformation [28].
Cholangiocarcinoma	Overexpression in patients’ cells; activation of the Akt/NF-κB and Wnt/β-catenin pathways [29]
Colorectal	Overexpression in patients’ cells and RARγ knockdown increased sensitivity to chemotherapeutics. [30]; vitamin A metabolism signature for brain metastasis [31]
Head and neck	Activation of the epidermal growth factor receptor and downstream Akt, Erk, Src, and YAP signalling [32]
Hepatocellular	Overexpression in patients’ cells; activation of the Akt/NF-κB pathways [33] and downregulation of E-cadherin for metastasis [34]
Ovarian	Overexpression in patients’ cells and cell line knockdown reduced xenograft growth [35]
Pancreatic	Overexpression in patients’ cells; chromatin epigenetic activation from histone H3 K27 acetylation [36,37]
Prostate	Overexpression in patients’ cells [38]; promotion of proliferation [39]; RARγ-Foxa1 promotion of progenitor cell identity/dedifferentiation [40].
Renal	Overexpression in patients’ cells [41]

**Table 2:** Targeting RARs directly to kill cancer cells.

Malignancy	Agent	Outcome from Antagonism of RARs
AML	Pan-RAR antagonist AGN193109	Delayed leukemogenesis in vivo and reduced stemness of Evi1^high^ AML cells [61]
Hepatocellular	Acacetin Inhibits RARγ transactivation	Apoptosis of cell line cells [62]
Pancreatic	RARγ antagonists LY2955303 MM11225	G1 arrest of cell line cells and reduced proliferation of pancreatic cancer organoids [37]
Paediatric brain	Pan-RAR antagonist AGN194310	Growth arrest and cell death of patients’ cells and the prevention of neurosphere formation [63]
Prostate	Pan-RAR antagonist AGN194310 RARγ antagonist AGN205728	G1 arrest and necroptosis of cell line and patients’ cells [39,64,65]
